# Functional–Taxonomic Scaling Resolves Conflicting Average Genome Size Estimates Across Environmental Gradients

**DOI:** 10.1002/advs.77591

**Published:** 2026-09-27

**Authors:** Huanhuan Zhu, Xiaoye Chen, Qiushi Li, Chenyu Sun, Haihua Xia, Yingxin Huang, Cong Wang, Peilin Chen, Cheng Gao

**Affiliations:** ^1^ State Key Laboratory of Microbial Diversity and Innovative Utilization Institute of Microbiology Chinese Academy of Sciences Beijing China; ^2^ College of Life Sciences University of Chinese Academy of Sciences Beijing China; ^3^ College of Life Sciences Shandong Agricultural University Taian China; ^4^ Innovation Research Center for Microbial Pharmacology Institute of Microbiology Heilongjiang Academy of Sciences Harbin China; ^5^ State Key Laboratory of Black Soils Conservation and Utilization Northeast Institute of Geography and Agroecology Chinese Academy of Sciences Changchun China

**Keywords:** alkaline gradient, average genome size, broad pH spectrum, genomic traits, methodological bias, microbial adaptation, saline gradient

## Abstract

Community‐level average genome size (AGS) is a key trait linking microbial diversity, functional potential, and eco‐evolutionary strategy, yet estimates of AGS diverge markedly among common methods. Here, we introduce and validate a framework that adjudicates conflicting AGS estimates by testing which estimate yields a gap between scaled functional diversity and scaled taxonomic diversity that is consistent with the expected positive association between genome size and gene functional breadth. Across independent environmental gradients, metagenomic AGS estimates align with this framework and expectations from metagenome‐assembled genome (MAG) dynamics and gene co‐occurrence network module sizes, whereas 16S rRNA metabarcoding‐based AGS estimates deviate, particularly in extreme environments with sparse reference coverage. Applying this validated framework, we reveal a U‐shaped relationship between soil pH and AGS across a broad pH range, unifying prior findings of decreasing AGS from acidic to neutral soils with a newly observed increase along an alkaline gradient. Applying the same framework to a saline‐gradient dataset, we further suggest that conclusions of a recent study regarding eco‐evolutionary trade‐offs under salt stress may stem from method biases. This work identifies a potentially pervasive conflict in AGS estimation, provides a framework to resolve it, and clarifies and expands relationships between AGS and the environment.

## Graphical abstract

Based on genome size‐functional breadth theory, we introduce and validate a framework that adjudicates conflicting average genome size (AGS) estimates by testing which estimate yields a gap between scaled functional diversity and scaled taxonomic diversity that is consistent with the expected positive association between genome size and gene functional breadth.

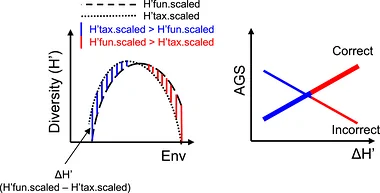

## 1 Introduction

Average genome size (AGS) has become a pivotal community‐level trait in microbial ecology, serving as a proxy for functional versatility and adaptation to environmental gradients across biomes [1, 2, 3, 4, 5, 6, 7]. Recent studies have robustly linked AGS to eco‐evolutionary processes, with clear associations to environmental filtering and shifts in ecological strategies along gradients of resource, stress, and disturbance [8, 9, 10, 11, 12, 13]. Genome size is relevant to the eco‐evolutionary strategies and environmental adaptation of both bacteria and fungi, but fungal AGS remains far less well developed than bacterial AGS [14, 15]. This limitation largely reflects the sparse and uneven coverage of fungal reference genomes and the typically low proportion of fungal reads in shotgun metagenomic datasets [16]. Accordingly, the present study focuses on bacterial AGS while acknowledging that fungal AGS represents an important but still underexplored knowledge gap.

AGS can be estimated directly from shotgun metagenomes, typically via detection of essential single‐copy genes (e.g., MicrobeCensus [17]), or indirectly by projecting community profiles (mostly from 16S rRNA gene metabarcoding sequencing) onto genome‐size reference catalogs such as the Genome Taxonomy Database (GTDB) [18]. Both approaches are widely used, but their concordance is not guaranteed. Discrepancies might arise from differences in reference database coverage, sequencing depth, taxonomic resolution, and contamination [19, 20, 21, 22, 23]. For example, because current reference genome databases are biased toward cultured and well‐studied lineages [20], often from neutral‐pH environments, metabarcoding‐based estimates of average genome size may be less reliable in extreme environments such as alkaline or saline systems. In contrast, shotgun metagenomic approaches can estimate community‐level average genome size without relying on taxon‐specific genome‐size references, making them particularly useful for underrepresented environments.

Soil pH and salinity are essential abiotic drivers of bacterial diversity, community structure and function [24, 25, 26, 27, 28, 29, 30, 31]. Recent work has extended these patterns to community‐level AGS, with most studies reporting AGS decreases from acidic toward neutral pH [10, 32, 33, 34, 35, 36]. However, two major gaps remain: (1) most AGS studies emphasize acidic to neutral soils [10, 32, 33, 34, 35, 36], leaving alkaline systems (pH > 7) underexplored; and (2) AGS responses to salinity are inconsistent in literatures, with increases, decreases, or no change reported depending on ecosystem and method [37, 38, 39, 40].

**FIGURE 1:**
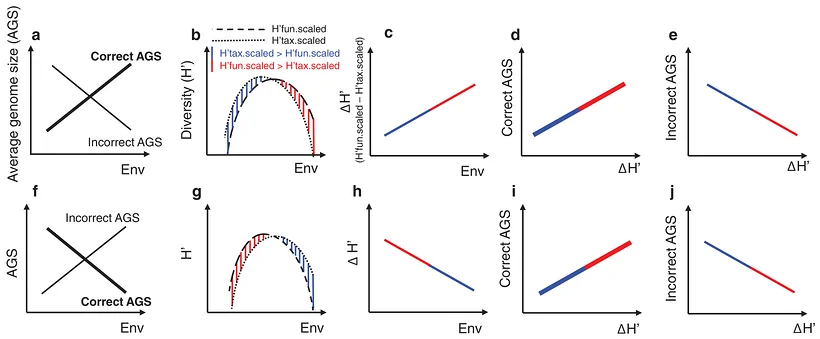
Framework for resolving conflicting average genome size (AGS) estimates along an environmental gradient. (a–e) Scenario where the true AGS increases along the gradient. (a) Two contradictory AGS trends from different estimation methods along the same gradient: correct AGS increases, whereas an incorrect estimate decreases. (b) Functional and taxonomic diversity are quantified as Shannon diversity using gene and taxon read abundances, respectively, and then rescaled to the range [0, 1] to obtain scaled functional diversity (H′fun.scaled, long‐dashed curve) and scaled taxonomic diversity (H′tax.scaled, dotted curve). Their difference, ΔH′ = H′fun.scaled − H′tax.scaled, provides a diversity‐based diagnostic of AGS trends. Under the assumption that increasing AGS elevates functional diversity conditional on taxonomic diversity, ΔH′ is expected to be negative at the low end and positive at the high end. A hump‐shaped diversity–gradient relationship is illustrated here, but the diagnostic logic also applies to other relationships, including U‐shaped patterns (Figure S1). (c) Expected increase in ΔH′ along the gradient. (d, e) Predicted relationships between ΔH′ and AGS estimates. The correct AGS estimate is expected to show a positive association with ΔH′ (d), whereas the incorrect estimate is expected to show no such positive association (e). (f–j) Scenario in which the true AGS decreases along the gradient. (f) Two AGS estimation approaches again produce opposing trends: the correct estimate decreases with the environmental gradient, whereas an incorrect estimate increases. (g) Conceptual relationship between diversity and the gradient. Under the assumption that decreasing AGS lowers functional diversity conditional on taxonomic diversity, ΔH′ is expected to be positive at the low end and negative at the high end. (h) Expected decrease in ΔH′ along the gradient. (i, j) Predicted relationships between ΔH′ and AGS estimates. The correct AGS estimate should remain positively associated with ΔH′ (i), whereas the incorrect estimate should not show the expected positive association (j).

Here, we introduce a framework to assess the reliability of conflicting AGS estimates by testing which estimate is more consistent with the expected positive association between genome size and functional breadth [3, 41]. Specifically, gene functional diversity was calculated as Shannon diversity based on gene read abundances within each sample, and the resulting values were then min–max rescaled to the range [0, 1] across all samples, allowing direct comparison with similarly scaled bacterial taxonomic diversity (Figure 1 and Figure S1). Under this framework, the more reliable AGS estimate is expected to produce a gap between scaled functional diversity and scaled taxonomic diversity that is consistent with the predicted contribution of genome size to functional breadth (Figure 1 and Figure S1).

We applied this framework to two independent datasets: a newly collected dataset spanning an alkalinity gradient and a public dataset spanning a salinity gradient [40]. In both datasets, we found that AGS estimates conflicted between shotgun metagenomes and 16S rRNA metabarcoding. We also generated metagenome‐assembled genomes (MAGs) and gene co‐occurrence networks to cross‐validate our adjudication of AGS estimates.

## 2 Results and Discussion

### 2.1 A Functional–Taxonomic Scaling Framework for Adjudicating Conflicting AGS Estimates

When different methods yield contradictory AGS estimates, we test which one is more plausible by asking whether it reconstructs the expected scaling between the diversity of gene functional potential and community taxonomic diversity along the environmental gradient. Empirically, larger genomes encode broader functional repertoires [3, 41]. Therefore, conditional on the observed taxonomic diversity, we expect functional diversity to increase with AGS, producing a functional–taxonomic diversity gap that co‐varies coherently with AGS.

Let H′tax and H′fun denote bacterial taxonomic diversity and functional gene diversity, respectively, both calculated as Shannon diversity indices from gene/taxon abundance data rather than presence/absence data. We rescale each within the gradient to [0, 1] to obtain H′tax.scaled and H′fun.scaled, and define the functional–taxonomic diversity gap as:

$$\Delta H^{\prime }=H^{\prime }\mathrm{fun}.\mathrm{scaled}-H^{\prime }\mathrm{tax}.\mathrm{scaled}$$

Under the null scenario where AGS does not vary along the environmental gradient, functional diversity is expected to track taxonomic diversity because a more taxonomically diverse community generally supports a broader functional gene repertoire. After rescaling both diversities to [0, 1], this coupling implies that the functional–taxonomic gap, ΔH′ = H′fun.scaled − H′tax.scaled, should remain close to zero and show little systematic change along the gradient.

By contrast, if AGS truly changes along the gradient, H′fun.scaled is expected to deviate from what would be predicted from H′tax.scaled alone. Because larger genomes tend to encode broader functional repertoires, an increase in AGS should manifest as a positive shift in ΔH′ (i.e., H′fun.scaled exceeding H′tax.scaled), whereas a decrease in AGS should manifest as a negative shift in ΔH′. Importantly, ΔH′ is defined purely from scaled taxonomic and functional diversity, so a positive association between ΔH′ and independently estimated AGS indicates that the inferred AGS variation is ecologically consistent with the expected coupling between genomic repertoire breadth and community‐level functional diversity. This correspondence is defined on scaled taxonomic and functional diversity and therefore should be robust to the specific shape of H′tax or H′fun along the gradient (e.g., linear or hump‐shaped).

Practically, consider two conflicting AGS series that vary in opposite directions with the environment (Figure 1a). Even when microbial diversity varies nonlinearly with the environment, such as in a hump‐shaped pattern, the “correct” AGS series should yield a ΔH′ that tends to increase where AGS increases and decrease where AGS decreases (Figure 1). Along the environmental axis, samples at the lower‐AGS end should exhibit smaller ΔH′, whereas those at the higher‐AGS end should exhibit larger ΔH′, producing a directionally consistent monotone trend of ΔH′ with AGS (Figure 1). The “incorrect” AGS estimate should fail to recover this expected positive ordering, often producing a weak, absent, or opposite ΔH′–AGS relationship (Figure 1). In summary, the “correct” AGS estimate is the one that yields a ΔH′–AGS relationship coherent with the expected positive association between genome size and functional breadth (Figure 1). In the following, we applied this approach to two independent datasets.

### 2.2 AGS Under Alkalinity: Increase in Metagenomes, Decrease in Metabarcoding

**FIGURE 2:**
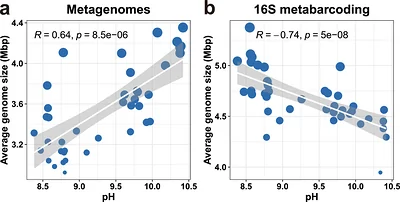
Method‐dependent associations between average genome size (AGS) and soil pH along alkaline gradients. (a) Shotgun metagenomes analyzed with MicrobeCensus show a positive correlation between soil pH and AGS. (b) 16S rRNA gene metabarcoding mapped to GTDB reference genomes, with AGS estimated by assigning ASV representatives to genomes and weighting by ASV relative abundance, indicates a negative correlation between pH and AGS. In both panels, point size reflects AGS estimated by the corresponding method.

Our alkalinity gradient dataset comprises shotgun metagenomic and 16S rRNA gene metabarcoding data from sodic alkaline soils (pH 8.5–10.5) of the Songnen Plain, Northeast China. Metagenomic analysis of prokaryotic reads (excluding viral and eukaryotic reads [10]) using MicrobeCensus [17] revealed a strong positive correlation between AGS and pH (Figure 2a), indicating larger AGS in more alkaline soils. In contrast, AGS inferred from 16S rRNA gene metabarcoding profiles mapped to GTDB genomes showed a strong negative correlation with pH (Figure 2b), highlighting method‐dependent directionality.

**FIGURE 3:**
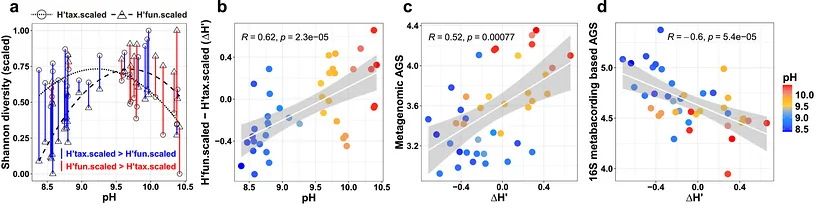
Discriminating the reliability of average genome size (AGS) estimates from metagenomes vs. metabarcoding. (a) Taxonomic diversity (H′tax.scaled; dotted curve) and functional gene diversity (H′fun.scaled; long‐dashed curve) each show unimodal responses to pH. Vertical segments depict ΔH′ = H′fun.scaled − H′tax.scaled, colored blue when ΔH′ < 0 and red when ΔH′ > 0. ΔH′ is mostly negative at lower pH (≈8.5) and positive at higher pH (≈10.5). (b) Positive correlation between ΔH′ and soil pH. (c, d) ΔH′ is positively correlated with metagenomic AGS (c) but not with 16S‐derived AGS (d).

We applied the abovementioned framework (Figure 1) to evaluate the reliability of these two estimates. Both scaled functional gene diversity (H′fun.scaled) and scaled taxonomic diversity (H′tax.scaled) exhibited unimodal relationships with pH (Figure 3a). The ΔH′ capturing the gap of H′fun.scaled against H′tax.scaled was more negative at the lower pH end (pH ≈ 8.5) and more positive (pH ≈ 10.5) at the higher pH end, as a result ΔH′ positively correlated with soil pH (Figure 3b). Furthermore, aligning ΔH′ with AGS estimates with both approaches, we found positive correlations between ΔH′ with metagenomic AGS estimates (Figure 3c), and a negative correlation between ΔH′ with 16S rRNA metabarcoding‐based AGS estimates (Figure 3d). Based on our framework that “correct” AGS correlate positively with ΔH′, our analysis supports the metagenomic AGS estimates (Figures 1, 2, 3).

**FIGURE 4:**
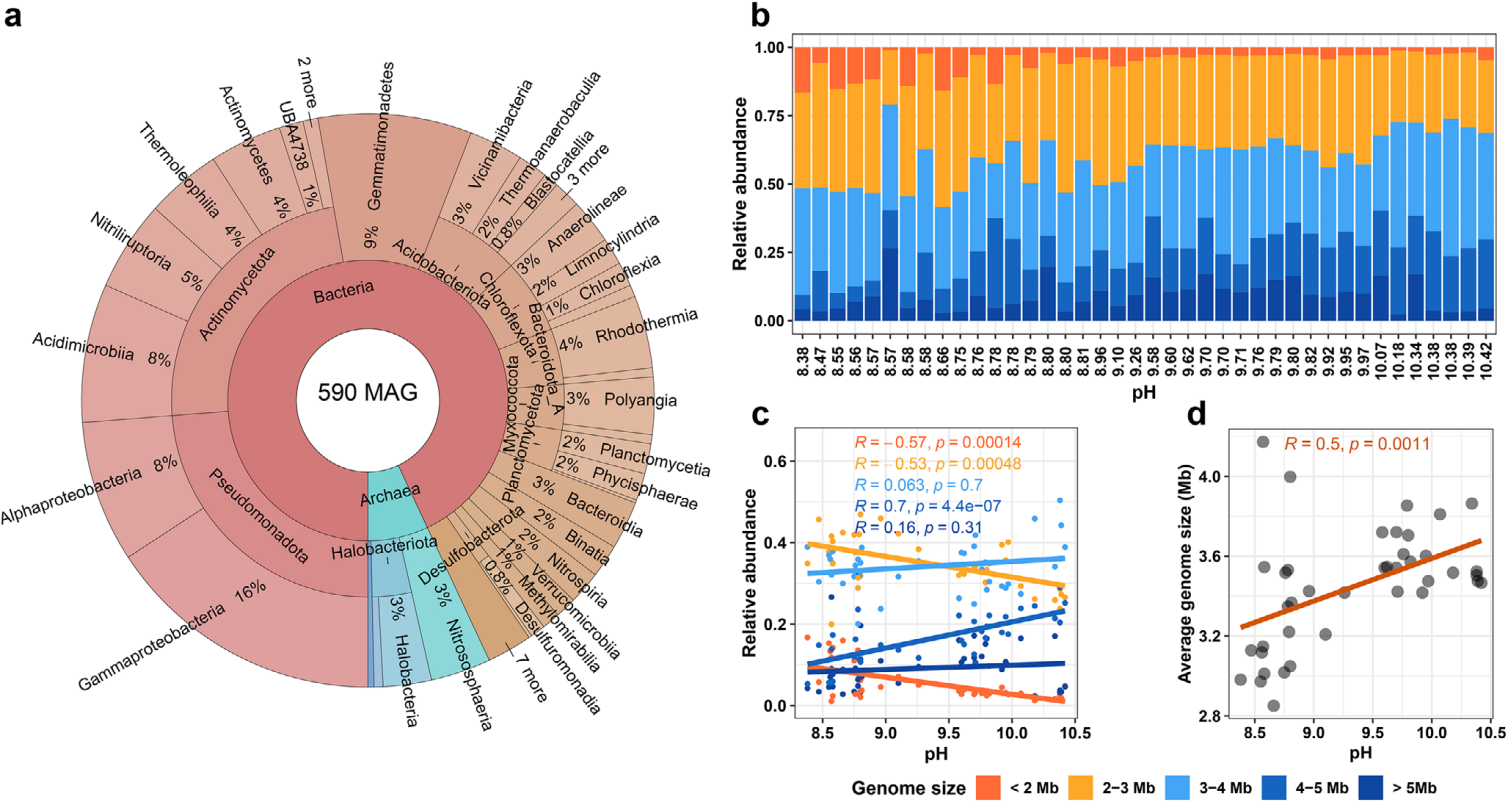
Metagenome‐assembled genomes (MAGs) cross‐validate adjudication of conflicting average genome size (AGS) estimates. (a) Overview of recovered MAGs. (b) Relative abundance of MAGs grouped by genome‐size class. (c) Smaller‐genome MAGs decrease, and larger‐genome MAGs increase in relative abundance with higher alkalinity. (d) MAG‐based AGS increases with alkalinity.

Metagenome‐assembled genomes (MAGs) can be used to cross‐validate the AGS estimates. Because the metagenomic AGS increases monotonically with alkalinity, larger‐genome MAGs are unlikely to show declines in relative abundance, and smaller‐genome MAGs are unlikely to show increases in relative abundance, as alkalinity rises. This pattern in metagenomic AGS is consistent with trends observed in MAGs. We recovered 590 high‐quality MAGs from the alkalinity dataset (Figure 4a). Grouping these MAGs by genome size showed that the correlation between alkalinity and relative abundance was negative for smaller genomes (< 2 Mb and 2–3 Mb) and positive for larger genomes (3–4 Mb, 4–5 Mb, and > 5 Mb) (Figure 4b,c). Taken together, the MAG‐based genome‐size patterns corroborate the metagenomic AGS trend of increasing genome size with higher alkalinity (Figure 4d).

**FIGURE 5:**
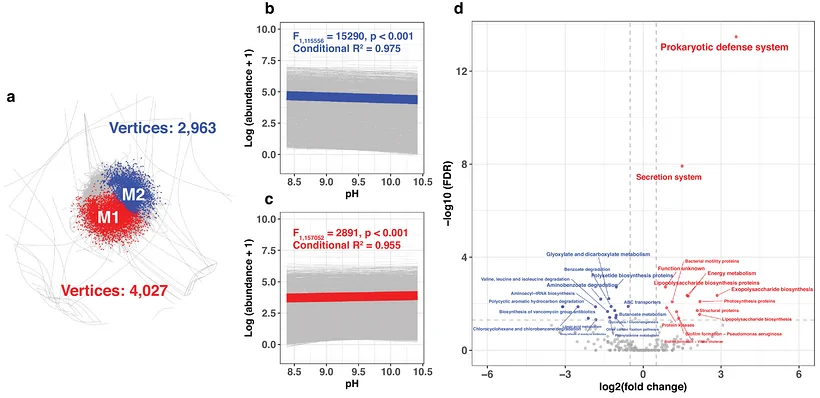
Gene co‐occurrence networks and environment–module associations. (a) Gene (KO) co‐occurrence network revealing two dominant modules: a larger hyperalkaline module (red; 4027 vertices) and a smaller near‐neutral pH module (blue; 2963 vertices). (b, c) Module–pH associations (linear mixed‐effects models, two‐sided tests). The relative abundance of genes in (b), the near‐neutral module (blue) decreases with soil pH, whereas the (c) hyperalkaline module (red) increases with soil pH. (d) KEGG pathway enrichment (level 3) for the two modules based on differential representation (two‐sided tests). The hyperalkaline module is enriched for signal transduction (kinase), energy metabolism, defense and secretion systems, cell motility, biofilm formation, and lipopolysaccharide and exopolysaccharide biosynthesis. The near‐neutral module is enriched for xenobiotics biodegradation, ABC transporters, carbohydrate metabolism, terpenoid and polyketide metabolism, amino acid metabolism, and secondary metabolite biosynthesis.

Genes located within the same genome tend to show correlated occurrence patterns across samples because they are co‐inherited and are therefore detectable together. Thus, AGS estimates can also be cross‐validated using gene co‐occurrence networks. Because metagenomic AGS increases monotonically with alkalinity, the hyperalkaline gene‐network module under higher‐AGS conditions would not likely contain fewer genes than the near neutral module. Consistent with this expectation, our gene co‐occurrence network analysis showed a much larger number of genes (4027 KEGG Orthology, KOs) positively correlated with soil pH, forming a larger hyperalkaline module, compared with fewer genes (2963 KOs) negatively correlated with soil pH, forming a smaller near‐neutral pH module (Figure 5). Building on this pattern described along the alkaline gradient, where more alkaline stressed, higher pH sites showed increased AGS together with a larger functional module, our results are conceptually consistent with prior work across an acid‐to‐neutral pH gradient [10]. In that study, larger AGS under more acidic stressed conditions coincided with a larger acid‐associated module (4309 KOs), whereas smaller AGS under neutral pH was accompanied by a smaller module (2777 KOs) [10]. Taken together, these observations across contrasting pH gradients suggest a general relationship in which AGS covaries positively with functional module size along environmental gradients. Functional enrichment analysis showed that the hyperalkaline module was most strongly enriched for defense mechanisms (log‐transformed fold change, logFC = 3.58), followed by biofilm formation (logFC = 1.29), cell motility (logFC = 1.12), and signal transduction (logFC = 0.91) (Figure 5). The enrichment of prokaryotic defense systems may protect genomes against phage attack and DNA damage under harsh conditions [42]. In parallel, biofilm formation and secretion‐related functions provide physical barriers and facilitate pH homeostasis and nutrient acquisition [43], while enhanced motility enables bacteria relocated to favorable microsites [44]. The enrichment of signal transduction components, including protein kinases, further suggests an enhanced capacity to sense and coordinate responses to fluctuating pH, osmotic, and oxidative stresses [3]. The prevalence of signal transduction is congruent with larger AGS in hyperalkaline soils and known links between genome size and signaling gene contents [3]. By contrast, the near neutral module was enriched for xenobiotics biodegradation, carbohydrate, terpenoid, polyketide, amino acid metabolism, and secondary metabolite synthesis (Figure 5), consistent with a more copiotrophic strategy under relatively milder, resource‐richer conditions [10]. Enrichment of xenobiotics biodegradation aligns with plant‐derived aromatics such as benzoate in these systems [45, 46].

**FIGURE 6:**
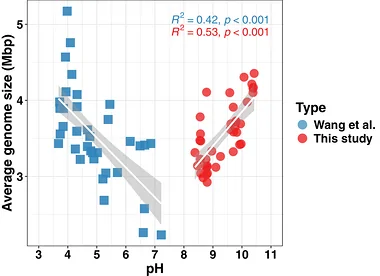
A unimodal relationship between soil pH and average genome size (AGS) across the broad soil pH spectrum (∼ 3.5–10.5), obtained by integrating the results of Wang et al. [10] (pH 3.5–7) and this study (pH 8.5–10.5; Figure 2a).

Whether the pH–AGS association is positive or negative under alkaline conditions is pivotal for inferring the shape of the pH–AGS relationship across the full pH spectrum. Previous studies have demonstrated a negative correlation between pH and AGS from acidic to neutral ranges [10, 32, 33, 34, 35, 36]. Consequently, a positive association in the alkaline range predicts a U‐shaped relationship across acidic, neutral, and alkaline pH, whereas a negative association would predict a broadly linear, uniformly negative trend. As our analysis indicates a positive association in alkaline systems, combined with the previously reported negative association at acidic–neutral pH [10, 32, 33, 34, 35, 36], this supports a U‐shaped pH–AGS relationship across the broad pH gradient (Figure 6). Notably, unlike our alkaline‐gradient dataset, in which metagenome‐ and 16S‐based approaches yielded divergent AGS estimates, previous studies along acidic–neutral pH gradients reported broadly consistent AGS patterns between metagenomic and 16S‐based analyses [10, 32, 33, 34, 35, 36]. This finding highlights the pivotal and nuanced role of soil pH in structuring microbiome genomic traits across broad environmental gradients, with implications for predicting microbial community function and resilience under global change.

**FIGURE 7:**
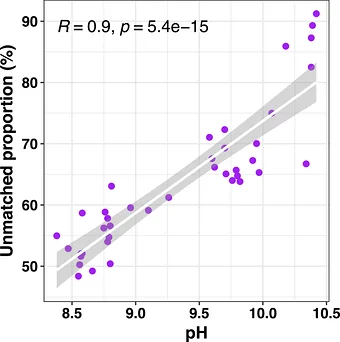
Potential sources of unreliability in 16S rRNA metabarcoding‐based average genome size (AGS) estimates. With mapping thresholds of > 99.5% identity and = 100% coverage, the proportion of GTDB‐unclassified bacterial taxa increases with soil pH, reaching > 90% in hyperalkaline samples, where AGS estimates from 16S rRNA metabarcoding appear less reliable.

We then explored potential sources of unreliability in 16S‐based AGS estimation. Along the pH gradient, the community relative abundance represented by taxa lacking genome‐size annotations increased substantially, reaching approximately 90% in hyperalkaline samples (Figure 7). This pattern indicates potential bias in indirect AGS estimation in environments containing substantial microbial novelty or lineages that are underrepresented in current genome databases. The particularly high proportion of unannotated reads in hyperalkaline samples may reflect both the limited research attention given to these extreme environments and the existence of yet‐to‐be‐characterized taxa and genetic diversity adapted to such conditions. To improve transparency, we recommend that future studies report, for each sample, the proportion of reads and taxa assigned to lineages with genome‐size annotations, the taxonomic rank at which genome‐size information was assigned, and the uncertainty associated with within‐taxon genome‐size variation.

Besides directly linking ASVs to GTDB genomes using stringent matching criteria, phylogeny‐based approaches provide a complementary strategy for estimating bacterial genome size, particularly for lineages lacking close genome representatives. Our application of a phylogeny‐based approach, implemented in genomesizeR [47], reduced the unannotated proportion from > 90% to ∼ 70% in hyperalkaline samples; however, the unannotated proportion still increased steeply along the alkalinity gradient (Figure S2). Consistent with the ASV–GTDB approach, the phylogeny‐based approach yielded a negative relationship between AGS and soil pH across the near‐neutral to alkaline range (Figure S2). Thus, the lack of reference genomes remains a major bottleneck for 16S‐based AGS inference in poorly annotated communities, and this limitation cannot be fully resolved by incorporating phylogenetic information alone.

The biased 16S AGS estimates, which stem from the limited availability of representative reference genomes from extreme environments, might be alleviated by adding MAGs as additional genome references. However, at present, most MAGs do not fully recover the ∼ 250‐bp 16S rRNA gene region that was amplified and used for ASV calling in this study. This makes it difficult to reliably link MAGs to the corresponding ASVs. Future studies using deeper sequencing and long‐read technologies to generate higher‐quality MAGs will be needed to address this limitation.

Incomplete reference coverage might not be the only explanation for the observed distortion. Taxonomy‐based AGS inference assumes that genome size is sufficiently conserved within the taxonomic units used for assignment [48], so that available reference genomes can serve as reliable proxies for taxa detected in environmental samples. This assumption may be weakened in extreme environments, where strong environmental selection can drive genome‐size divergence within related taxa or favor lineages with distinct genome‐size strategies [49]. As a result, AGS estimates may be biased not only when genome‐size annotations are missing, but also when available reference genomes do not capture the genome‐size distributions of taxa inhabiting extreme conditions. Therefore, taxonomy‐based AGS estimates may become inaccurate even for annotated taxa.

To explore this potential, we examined within‐species genome size variation across environmental gradients using publicly available global MAGs [50]. Species were defined as groups of high‐quality MAGs (completeness > 90% and contamination < 5%) sharing average nucleotide identity greater than 96%. Across the pH gradient, we analyzed 24 species represented by at least five MAGs. Seven of the 24 species showed significant correlations between genome size and pH: four exhibited decreasing genome size from acidic to neutral conditions, two exhibited increasing genome size from neutral to alkaline conditions, and one exhibited decreasing genome size from neutral to alkaline conditions (Figure S3). These results indicate that environment‐associated genome size variation may contribute to AGS variation in some taxa, in addition to shifts in community composition.

### 2.3 AGS Under Salinity: Increase in Metagenomes, Decrease in Metabarcoding

**FIGURE 8:**
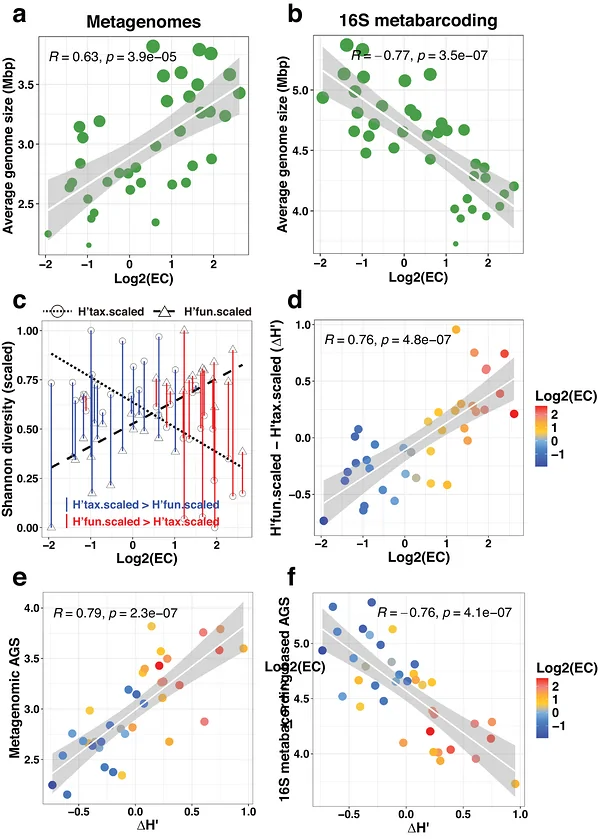
Results of re‐analysis of public salinity dataset. (a, b) Conflicting average genome size (AGS) estimation along a salinity gradient in Dongying. (a) Metagenomic AGS show a positive correlation with soil salinity (EC: electrical conductivity). (b) 16S‐GTDB AGS show a negative correlation with soil salinity. In both panels, point size reflects AGS estimated by the corresponding method. (c–f) Metagenomic AGS is reliable. (c) Taxonomic diversity (H′tax.scaled; dotted line) decreases, and functional gene diversity (H′fun.scaled; long‐dashed line) increases with rising salinity. (d) Positive correlation between ΔH′ and soil salinity. (e, f) ΔH′ is positively correlated with metagenomic AGS (e) but not with 16S‐derived AGS (f).

Analysis of a public saline gradient dataset [40] revealed a similar methodological divergence: metagenomic AGS increased with salinity, while 16S‐inferred AGS decreased (Figure 8a,b). Meanwhile, functional gene diversity increased, and taxonomic diversity decreased with rising salinity; consequently, the ΔH′ (H′fun.scaled − H′tax.scaled) increased with salinity (Figure 8c,d). Furthermore, aligning ΔH′ with AGS estimates with both approaches, we found positive correlations between ΔH′ with metagenomic AGS (Figure 8e), and negative correlation between ΔH′ with 16S AGS (Figure 8f). Based on our framework that “correct” AGS correlate with ΔH′ positively, our analysis supports the metagenomic AGS estimates (Figures 1, 2, 3). The fraction of unmapped taxa in 16S‐based projections accounted for ∼ 60%–90% of relative abundance, increasing strongly with salinity (Figure S4), further highlighting the unreliability of 16S‐based AGS inference in novelty‐rich, extreme environments.

We recovered 349 high‐quality MAGs from the salinity dataset (Figure S5). Consistent with the metagenomic AGS pattern, MAGs with larger genomes were positively correlated with salinity, whereas MAGs with smaller genomes were negatively correlated with salinity (Figure S5). The metagenomic AGS increase with salinity is also consistent with the gene co‐occurrence network: a substantially larger number of genes (3642 KOs) exhibited positive correlations with soil salinity, forming a larger, saline module, compared with fewer genes (2222 KOs) that exhibited negative correlations, forming a smaller, near‐non‐saline module (Figure S5). Functional enrichment indicated that the saline module was enriched for signaling and cellular community, whereas the near‐non‐saline module was enriched for translation and xenobiotics biodegradation (Figure S5).

Notably, in their original paper [40], the authors reported that bacterial genome size decreases and archaeal genome size increases with rising salinity. However, their analysis focused on only 500 taxa, representing a small fraction of total prokaryotic diversity [40]. Moreover, these 500 taxa are heavily biased toward less saline environments and account for as low as 1/8 of relative abundance in some saline samples [40]. Thus, their findings, which contrast with our reanalysis, are likely undermined by critical methodological shortcomings in taxon selection and data coverage.

Moving beyond alkalinity and salinity, future studies should further evaluate potential bias in AGS estimation across ecosystems where microbial reference genomes remain limited, such as extremely hot, cold, anaerobic, drought‐stressed, or otherwise under‐sampled environments [30]. At larger spatial scales, recent studies have frequently reported a decline in bacterial AGS from acidic toward neutral pH conditions, using both metagenome‐based and taxonomic‐inference approaches. This consistency suggests that broad‐scale AGS trends along well‐studied environmental gradients may be relatively robust.

Our study has limitations. The proposed framework was developed for prokaryotic communities, in which genome size is generally linked to gene repertoire and can therefore serve as an approximate proxy for functional potential [41]. However, this assumption may not be directly transferable to fungi. In fungal genomes, variation in genome size does not necessarily scale proportionally with protein‐coding gene content because of highly variable fractions of non‐coding DNA, repetitive elements, and other non‐genic regions [51]. In addition, fungal AGS estimation remains challenging because taxonomy‐based inference is limited by the sparse and uneven representation of fungal genomes in reference databases, whereas metagenome‐based inference is strongly constrained by the low proportion of fungal reads in shotgun metagenomic datasets [14, 16].

Our framework also assumes that the relationship between genome size and functional repertoire can be scaled from individual genomes to community‐level functional diversity. Although this relationship has been demonstrated for individual genomes [41] and supported by community‐level analyses along soil pH gradients from acidic to neutral conditions [10], a positive association between AGS and community‐level functional diversity is not guaranteed in other systems. This is because community functional diversity is shaped not only by the aggregate genomic potential of constituent taxa, but also by how functions are distributed among taxa [52]. For example, communities dominated by large‐genome organisms may exhibit substantial functional redundancy, whereas communities composed of small‐genome organisms may maintain high functional complementarity through metabolic specialization, cross‐feeding, syntrophy, or division of labor [53, 54]. These differences in redundancy and complementarity could make the relationship between AGS and community‐level functional diversity context dependent.

## 3 Conclusion

AGS is emerging as a pivotal genomic trait underpinning eco‐evolutionary of microbiome. Both metagenome and metabarcoding are widely used to estimated AGS, but they can yield inconsistent results. This study introduces a diagnostic to arbitrate conflicting AGS inferences and applied it to two independent gradients. The contrasting AGS estimation in these two independent gradients suggests this method‐dependent AGS estimation may be underappreciated in prior studies, and that cautions studies used only one approach (particularly taxa inference), especially in extreme environments. Our arbitrate approach is consistent with the genome size–functional breadth theory [3, 41], the AGS–MAG dynamic and AGS–module relationship, and applicable to system whether microbial diversity changed linearly or nonlinearly. Besides, as most studies to date have focused on acidic to neutral soils [10, 32, 33, 34, 35, 36], adding the new finding of this study, we can conclude a unimodal AGS–pH relationship across the broad soil pH spectrum (∼ 3.5–10.5) (Figure 6). Extending from soil pH to salinity, we clarify that AGS–salinity relationships are positive in soils when assessed by metagenomics, resolving previous inconsistencies in the literature [37, 38, 39, 40].

## 4 Materials and Methods

### 4.1 Sampling

This study sampled 40 soils from 8 locations (123°27′15″E–123°51′17″E, 44°31′16″N–45°36′37″N) in the Songnen Plain to form an alkaline pH gradient of 8.5–10.5 [55]. The 8 locations comprised one bare land site (pH 9.60–9.79), two sites vegetated by *Suaeda glauca* (pH 9.76–10.42), and five sites vegetated by maize (*Zea mays*) (pH 8.38–10.34) [55]. At each location, five samples, more than 20 m apart, were collected and sieved through a 2 mm mesh [55]. The sieved soil was immediately placed in resealable plastic bags. Part of the collected soil was transported on ice and stored at −80°C in the laboratory for DNA extraction and sequencing, while the remainder was air‐dried at room temperature for subsequent analysis of soil physicochemical properties.

### 4.2 Soil Physicochemical Properties

Soil total carbon, total nitrogen, total phosphorus, available phosphorus, available nitrogen, pH, electrical conductivity, and salinity were measured by Inner Mongolia Dingcheng Testing Services Co., Ltd. Total carbon and total nitrogen were determined using an elemental analyzer (Elementar, Germany). Total phosphorus was determined after alkali fusion using the molybdenum antimony ascorbic acid colorimetric method with a UV–vis spectrophotometer (PerkinElmer, USA). Available phosphorus was extracted with bicarbonate and measured using the same colorimetric method. Available nitrogen was measured by the alkaline hydrolysis–diffusion method using a Kjeldahl nitrogen analyzer (FOSS, Denmark). Soil pH was determined using a pH meter (Sartorius, Germany), with a soil‐to‐water ratio of 1:2.5 (m/v). Soil electrical conductivity was measured using a conductivity meter (Shanghai Yidian Scientific Instrument Co., Ltd., China) at a soil‐to‐water ratio of 1:5 (m/v), and salinity was measured by the gravimetric method.

### 4.3 DNA Extraction

Soil DNA were extracted using the DNeasy PowerSoil Pro Kit (Qiagen, Germany) following the manufacturer's instructions with slight modifications: after adding CD1 buffer, samples were incubated at 65°C for 10 min and homogenized with a FastPrep‐24 5G homogenizer (MP Biomedicals, USA) for 10 s at 6 m s^−^^1^. The concentration and purity of the extracted DNA were measured using a NanoDrop 2000 spectrophotometer (Thermo Fisher Scientific, USA), and DNA was stored at −20°C for subsequent use.

### 4.4 Metabarcoding Sequencing

The bacterial 16S rRNA gene was amplified using primers 515F (5′‐GTGYCAGCMGCCGCGGTAA‐3′) and 806R (5′‐GGACTACNVGGGTWTCTAAT‐3′) [56]. During PCR, a unique barcode sequence was added to the forward primer of each sample within a 96‐well plate to distinguish individual samples. The PCR reaction (20 µL) contained 10 µL 2× Phanta Flash Master Mix, 2.5 µL forward primer (10 µm), 2.5 µL reverse primer (10 µm), 2.5 µL DNA template (5 ng/µL), 0.5 µL ddH_2_O, and 2 µL 0.3% bovine serum albumin (BSA). PCR cycling conditions were: initial denaturation at 98°C for 30 s; 35 cycles of 98°C for 10 s, 56°C for 5 s, and 72°C for 40 s; and a final extension at 72°C for 1 min. After amplification, PCR products from each sample were pooled in equal volumes, purified using a gel extraction kit (Axygen, USA), and quantified on a Qubit 2.0 fluorometer (Life Technologies, USA). Sequencing was performed on an Illumina NovaSeq 6000 platform at Shanghai Personal Biotechnology Co., Ltd., China.

The 16S rRNA sequencing data were processed and quality‐controlled using QIIME 2 (v2024.5) [57]. Reads were demultiplexed by sample barcodes, and primers were trimmed with q2‐cutadapt plugin [57]. Low‐quality reads were filtered based on expected errors and a minimum Phred quality threshold of 25. Sequences were denoised and inferred as amplicon sequence variants (ASVs) at 100% similarity using QIIME2 DADA2 pipeline [57, 58]. Taxonomic classification of ASVs was performed using the Naïve Bayes classifier in the QIIME 2 q2‐feature‐classifier plugin [59], pre‐trained on the SILVA (v138.2) database [60, 61]. Community‐weighted genome size was estimated by aligning 16S rRNA gene metabarcoding sequences to the Genome Taxonomy Database (GTDB), which provides representative genome sizes for corresponding taxa [62, 63]. ASVs were retained for GTDB‐based genome size estimation only if they matched representative genomes at ≥ 99.5% identity and 100% coverage [64]. Alternatively, we estimated AGS using genomesizeR [47], which infers genome size based on phylogenetic placement of taxa.

### 4.5 Metagenomic Sequencing

Shotgun metagenome sequencing was performed on the DNBSEQ platform in PE150 mode at Shenzhen BGI Genomics Co., Ltd., China. Prior to sequencing, DNA was sheared into smaller fragments by ultrasonication, and paired‐end libraries were prepared with the MGIEasy Universal DNA Library Prep Set (MGI, Shenzhen, China). Raw reads were processed with SOAPnuke [65] to remove adapters, contaminants, and low‐quality bases. Reads were discarded if they showed contamination, were shorter than 150 bp, contained more than 0.1% ambiguous bases, or had more than 50% of bases with a quality score below 20. Clean reads were reported using the Phred+33 quality scoring system. In total, 40 metagenomic DNA samples yielded approximately 939.67 Gb of clean data (mean 24.09 Gb per sample).

To remove potential host‐derived sequences, reference genomes of the dominant plant species at each site were downloaded from NCBI. Specifically, the *Zea mays* reference genome (GCF_902167145.1_Zm‐B73‐REFERENCE‐NAM‐5.0) and the *Suaeda glauca* reference genome (GCA_043643395.1_ASM4364339v1) were obtained in FASTA format. Clean reads from each sample were aligned to the corresponding host reference genome using Bowtie2 (v2.5.1) [66, 67] to generate host‐filtered reads. High‐quality reads were then assembled into contigs using MEGAHIT (v1.2.9) [68]. Assemblies were performed with multiple k‐mer sizes (–k‐list 21, 29, 39, 59, 79, 99, 119, 141) to optimize assembly quality. To focus on prokaryotes, we employed two complementary strategies. First, we identified and removed eukaryotic and viral contigs from assembled contigs using EukRep (v0.6.7) [69] and VIBRANT (v1.2.1) [70]. Second, we identified prokaryotic contigs by classifying the assembled contigs against the NCBI nucleotide (core_nt) database (updated October 15, 2025) using Kraken2 [71], retaining contigs assigned to Bacteria (taxid: 2) and Archaea (taxid: 2157). Contigs from these two strategies are used for the following pipeline, respectively, and notably these two strategies generated highly consistent results (Figure S6). The Redundans pipeline was applied to reduce redundancy among assembled contigs, using an overlap threshold of 80% and a sequence identity cutoff of 90% [72]. The resulting nonredundant contigs were used for gene prediction with Prodigal (v2.6.3) [73]. CD‐HIT (v4.8.1) was then applied to remove redundancy among predicted genes, using a 95% sequence identity threshold and 90% alignment coverage for the shorter sequence [74]. Gene abundance in each sample was quantified with Salmon (v1.6.0) by mapping raw reads to the nonredundant gene catalog while accounting for gene length and read count [75]. Functional assignments were performed with EggNOG‐mapper against the eggNOG database, including the identification of KO terms [76]. The average genome size of prokaryotic communities in each sample was estimated with the MicrobeCensus pipeline, which leverages coverage of ∼30 universally conserved single‐copy genes in bacteria and archaea [17]. Because these core genes constitute a larger fraction of smaller genomes, higher coverage of the marker set indicates a smaller average genome size. Prokaryotic AGS was calculated using bacteria‐ and archaea‐specific reads that recruited and extracted from the quality‐filtered metagenomic read pool by using BBMap (v39.01) (https://github.com/BioInfoTools/BBMap/). Metagenome‐assembled genomes (MAGs) were reconstructed using CONCOCT, MaxBin2, and MetaBAT2 within metaWRAP v1.3.2 [77], refined with dRep v3.4.2 [78], quality assessed with CheckM v1.2.2 [79], and taxonomically classified using GTDB‐Tk v2.1.1 [80].

### 4.6 Statistical Analyses

Data analysis and visualization were conducted in R v4.5.0 [81]. To examine how AGS estimated by two methods varied along the pH gradient, Spearman's correlation tests were performed with *p* values adjusted using the false discovery rate (FDR) method. To investigate how taxonomic and functional gene diversity change with increasing pH, the diversity function in the vegan package [82] was first used to calculate both the 16S ASV Shannon index and the metagenome KO Shannon index. Quadratic regression models were fitted using the lm function (Shannon index ∼ pH + I(pH^2)). A linear regression model was used to examine the association between microbial taxonomic diversity (16S ASV Shannon index) and functional gene diversity (metagenome KO Shannon index). For the metabarcoding dataset, the relative abundance of ASVs without corresponding genome size information (“unmatched ASVs”) was calculated, and its relationship with soil pH was assessed using Spearman's correlation.

To construct a co‐occurrence network of KOs, KOs with zero counts or low prevalence (present in fewer than 20 samples) were first removed. Pairwise Spearman correlation using the psych package [83] was applied to identify KO pairs with correlation > 0.6 and FDR‐adjusted *p* < 0.05. These pairs were used to build a network with the igraph package [84]. The co‐occurrence network comprises two major modules: module 1 (M1; 4,027KOs) and module 2 (M2; 2,963 KOs), which were detected by using the cluster_fast_greedy function in the igraph package [84]. The relationship between soil pH and vertices in the two modules was analyzed using the lme function in the nlme package [85]. To compare functional differences between the two modules, differential abundance analysis of KOs mapped to KEGG pathways was performed with the edgeR package [86]. KOs with |log2 fold change| ≥ 0.5 and FDR < 0.05 were considered significantly differentially abundant. Volcano plots were generated with ggplot2 [87], highlighting significantly enriched KOs.

### 4.7 Public Dataset

The public saline dataset was published by Dong et al. [40]. A total of 37 soil samples along a salinity gradient were collected in Dongying, China, and subjected to shotgun metagenomic sequencing and 16S rRNA gene metabarcoding [40]. These data were analyzed using bioinformatic and statistical workflows similar to those described above for our alkaline dataset.

The public global MAGs were published by Ma et al. [50]. A total of 40,039 MAGs were reconstructing from 3304 global soil metagenomes. Paired average nucleotide identity (ANI) of high‐quality MAGs (completeness > 90% and contamination < 5%) was calculated using skani v0.3.2 (default parameters adjusted with –min‐af 0.6 and –medium mode) [88]. MAG pairs with an ANI exceeding 96% were clustered to define distinct taxonomic units representing species [89]. Species comprising more than four MAGs and distributed across soils with a pH range or a salinity (electrical conductivity) range greater than 0.5 units were retained, resulting in a final dataset of 24 species represented by 293 MAGs. Soil pH and salinity (electrical conductivity) values for each MAG were extracted from SoilGrids 2.0 [90] and Wang et al. [91] based on the latitude and longitude coordinates provided by Ma et al. [50].

## Author Contributions

C.G., P.C., H.Z., and C.X. conceived and designed the study. C.G., P.C., H.Z., Y.H., and H.X. collected the alkaline samples. C.X. and H.Z. performed the molecular analyses; C.X., P.C., Q.L., and H.Z. analyzed the data. C.G., P.C., C.X., and H.Z. wrote the manuscript. All authors discussed approaches, results, and approved the final manuscript.

## Conflicts of Interest

The authors declare no conflicts of interest.

## Supporting information




**Supporting File**: advs77591‐sup‐0001‐SuppMat.pdf.

## Data Availability

All 16S metabarcoding (PRJCA052504) and metagenomic data (PRJCA052803) are available in National Genomics Data Center database. All metagenome‐assembled genomes (MAGs) generated in this study have been deposited in Zenodo (https://doi.org/10.5281/zenodo.22037407). The source code implementing the analyses in this manuscript is available on GitHub (https://github.com/hhzhu‐0185/Estimates‐of‐Average‐Genome‐Size‐Across‐Environmental‐Gradients).
