# Supplementary material for: Functional–Taxonomic Scaling Resolves Conflicting Average Genome Size Estimates Across Environmental Gradients
Source: Adv Sci (Weinh). 2026 Sep 27:e77591. Online ahead of print. doi: 10.1002/advs.77591 (PMC13616379; doi:10.1002/advs.77591)
Supplement: Supplementary file 1 [file ADVS-9999-e77591-s001.pdf]

Supplementary Materials for

Functional–Taxonomic Scaling Resolves Conflicting Average Genome Size Estimates Across Environmental Gradients

Huanhuan Zhu[#], Chen Xiaoye[#], Qiushi Li, Chenyu Sun, Haihua Xia, Yingxin Huang, Cong Wang, Peilin Chen*, Cheng Gao*

[#] These authors contributed equally to this work.

* Corresponding author (Peilin Chen: chenpl@im.ac.cn; Cheng Gao: gaoc@im.ac.cn)

This PDF file includes:

Figure S1-S6

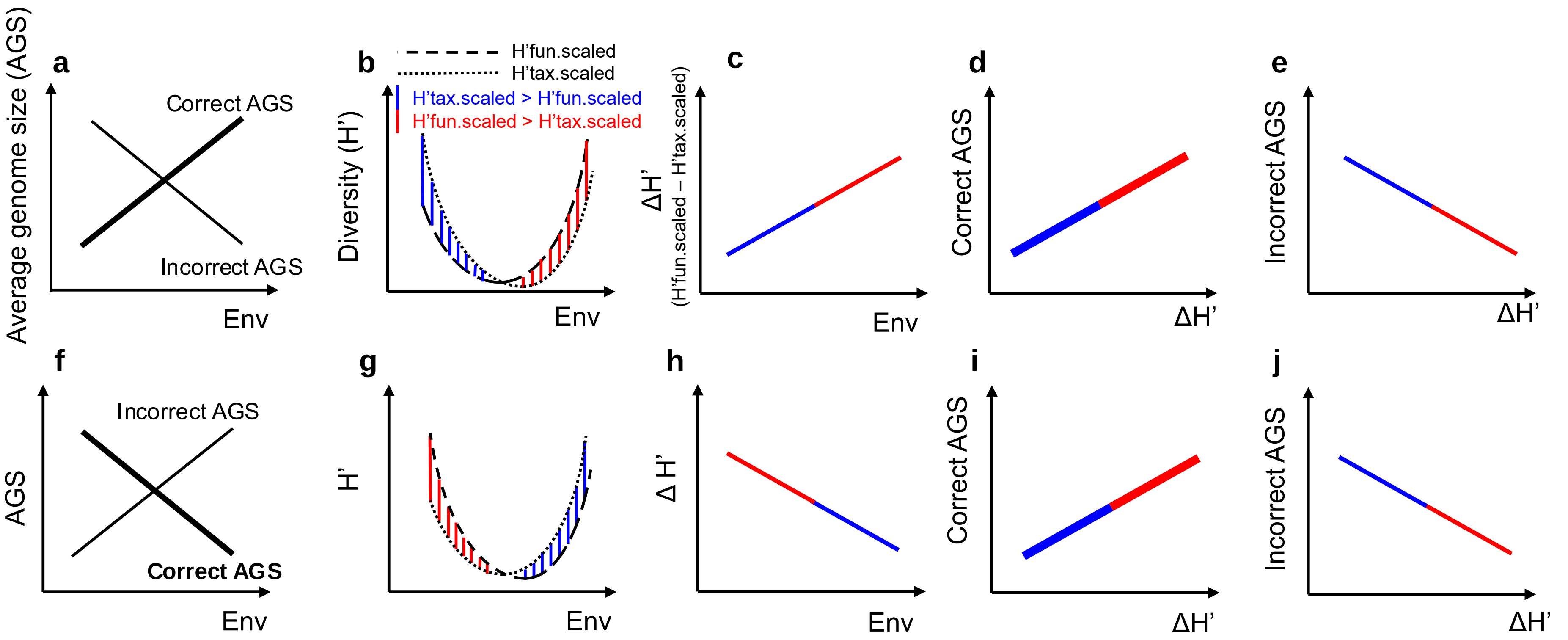

Figure S1 | Framework to diagnose conflicting AGS estimates when diversity exhibits U-shaped patterns. This figure is complementary to Figure 1.

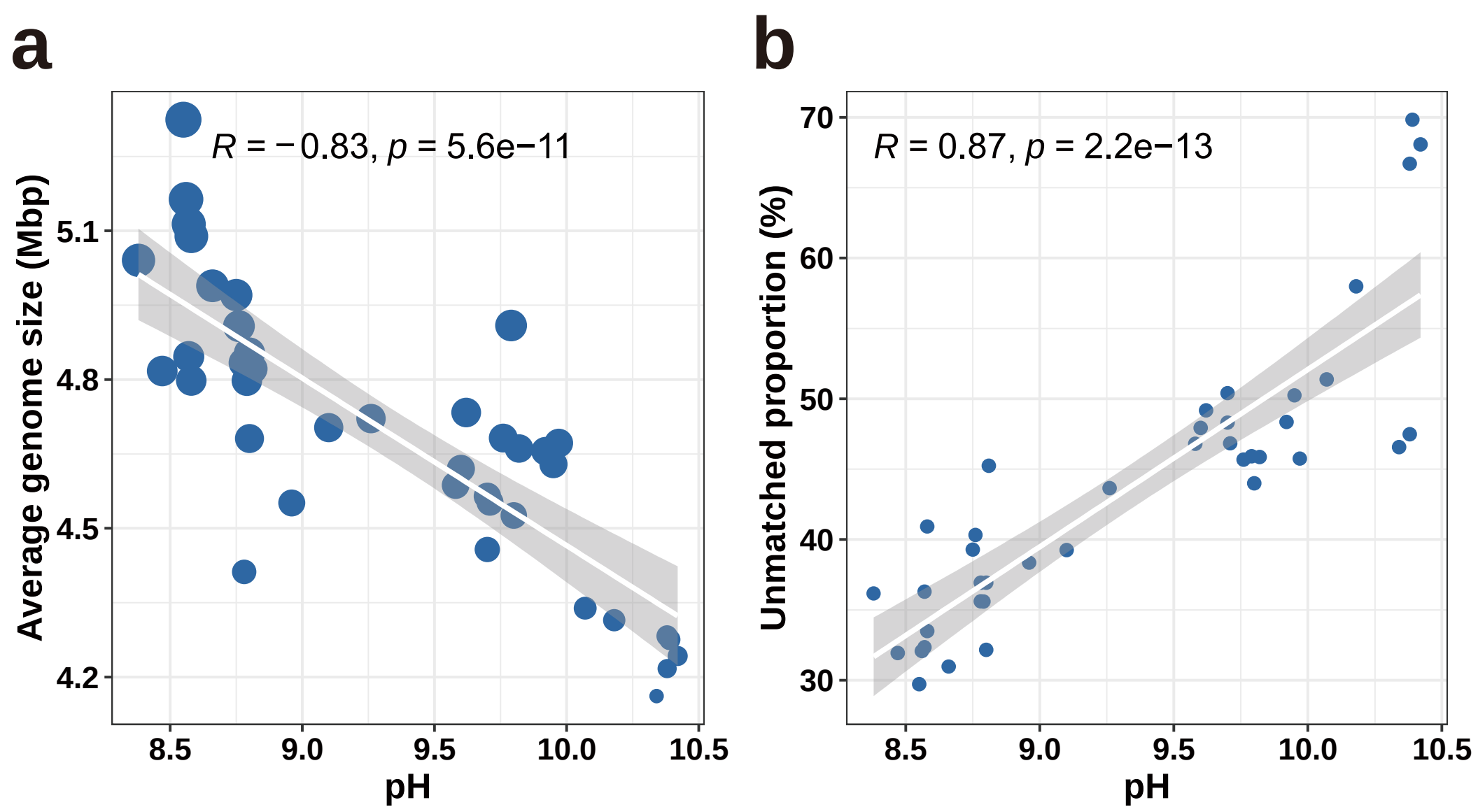

Figure S2 | Phylogeny-based estimation of average genome size (AGS). **a**, AGS estimated using the phylogeny-based approach implemented in genomesizeR showed a negative correlation with soil pH across the near-neutral to hyperalkaline range. **b**, The proportion of unannotated reads used for AGS estimation increased sharply along the soil pH gradient from near-neutral to hyperalkaline conditions.

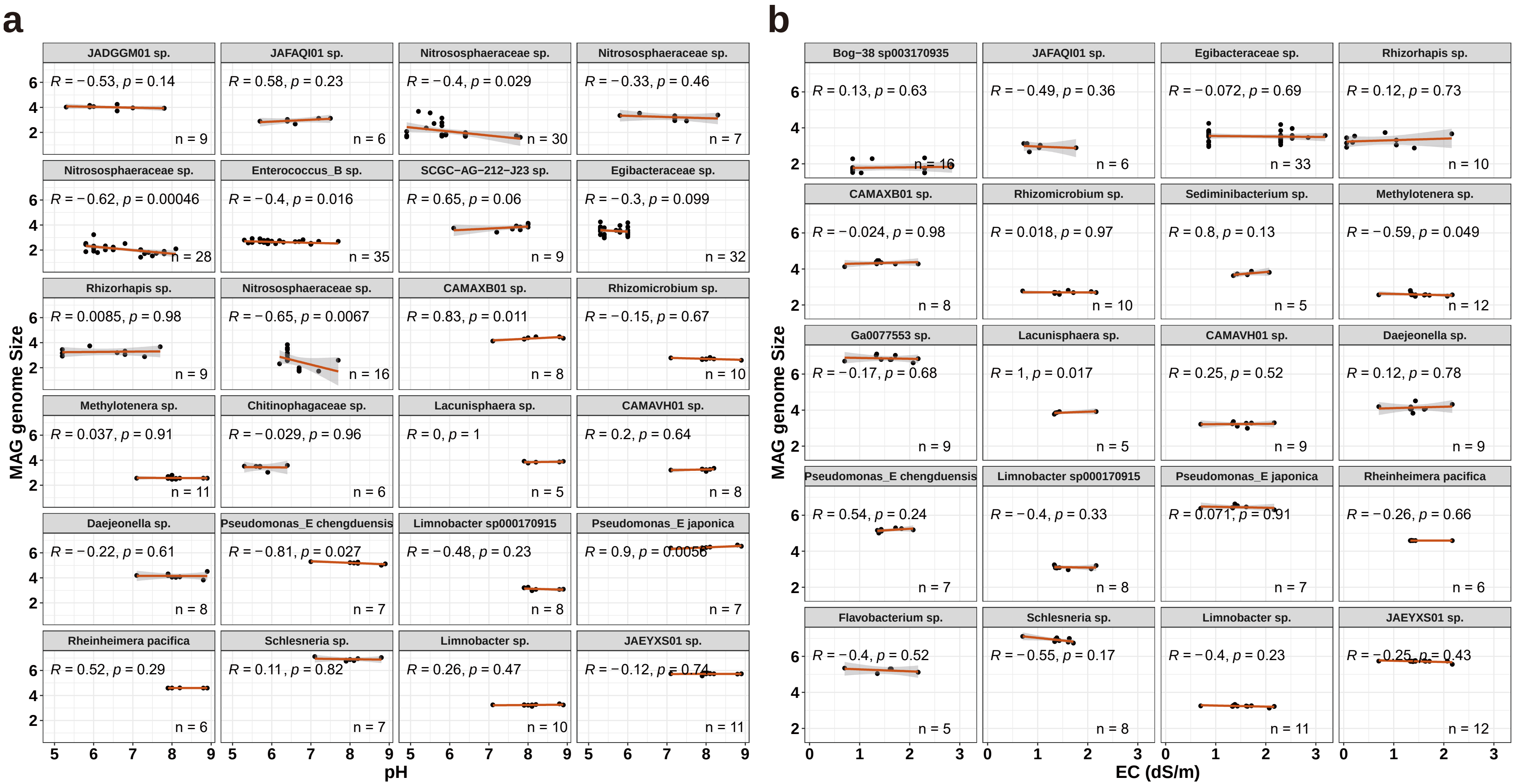
a
JADGGM01 sp.
R = −0.53, p = 0.14
n = 9
JAFAQI01 sp.
R = 0.58, p = 0.23
n = 6
Nitrososphaeraceae sp.
R = −0.4, p = 0.029
n = 30
Nitrososphaeraceae sp.
R = −0.33, p = 0.46
n = 7
Nitrososphaeraceae sp.
R = −0.62, p = 0.00046
n = 28
Enterococcus_B sp.
R = −0.4, p = 0.016
n = 35
SCGC−AG−212−J23 sp.
R = 0.65, p = 0.06
n = 9
Egibacteraceae sp.
R = −0.3, p = 0.099
n = 32
Rhizorhapis sp.
R = 0.0085, p = 0.98
n = 9
Nitrososphaeraceae sp.
R = −0.65, p = 0.0067
n = 16
CAMAXB01 sp.
R = 0.83, p = 0.011
n = 8
Rhizomicrobium sp.
R = −0.15, p = 0.67
n = 10
Methylotenera sp.
R = 0.037, p = 0.91
n = 11
Chitinophagaceae sp.
R = −0.029, p = 0.96
n = 6
Lacunisphaera sp.
R = 0, p = 1
n = 5
CAMAVH01 sp.
R = 0.2, p = 0.64
n = 8
Daejeonella sp.
R = −0.22, p = 0.61
n = 8
Pseudomonas_E chengduensis
R = −0.81, p = 0.027
n = 7
Limnobacter sp000170915
R = −0.48, p = 0.23
n = 8
Pseudomonas_E japonica
R = 0.9, p = 0.0056
n = 7
Rheinheimera pacifica
R = 0.52, p = 0.29
n = 6
Schlesneria sp.
R = 0.11, p = 0.82
n = 7
Limnobacter sp.
R = 0.26, p = 0.47
n = 10
JAEYXS01 sp.
R = −0.12, p = 0.74
n = 11
MAG genome Size
pH
b
Bog−38 sp003170935
R = 0.13, p = 0.63
n = 16
JAFAQI01 sp.
R = −0.49, p = 0.36
n = 6
Egibacteraceae sp.
R = −0.072, p = 0.69
n = 33
Rhizorhapis sp.
R = 0.12, p = 0.73
n = 10
CAMAXB01 sp.
R = −0.024, p = 0.98
n = 8
Rhizomicrobium sp.
R = 0.018, p = 0.97
n = 10
Sediminibacterium sp.
R = 0.8, p = 0.13
n = 5
Methylotenera sp.
R = −0.59, p = 0.049
n = 12
Ga0077553 sp.
R = −0.17, p = 0.68
n = 9
Lacunisphaera sp.
R = 1, p = 0.017
n = 5
CAMAVH01 sp.
R = 0.25, p = 0.52
n = 9
Daejeonella sp.
R = 0.12, p = 0.78
n = 9
Pseudomonas_E chengduensis
R = 0.54, p = 0.24
n = 7
Limnobacter sp000170915
R = −0.4, p = 0.33
n = 8
Pseudomonas_E japonica
R = 0.071, p = 0.91
n = 7
Rheinheimera pacifica
R = −0.26, p = 0.66
n = 6
Flavobacterium sp.
R = −0.4, p = 0.52
n = 5
Schlesneria sp.
R = −0.55, p = 0.17
n = 8
Limnobacter sp.
R = −0.4, p = 0.23
n = 11
JAEYXS01 sp.
R = −0.25, p = 0.43
n = 12
MAG genome Size
EC (dS/m)

Figure S3 | Within-species genome size variation across soil pH and salinity gradients. A species was defined as a group of metagenome-assembled genomes (MAGs) from a public global MAG dataset [1] that shared > 96% average nucleotide identity (ANI). **a**, Across the soil pH gradient, within-species genome size variation was significantly negatively correlated with pH in five species (four across the acid-to-neutral range and one across the neutral-to-alkaline range), significantly positively correlated with pH in two species across the neutral-to-alkaline range, and not significantly correlated with pH in 17 species. In total, 24 species were examined, each represented by at least five MAGs. **b**, Across the soil salinity gradient, within-species genome size variation was significantly correlated with salinity (electrical conductivity (EC)) in two of the 20 examined species, each represented by at least five MAGs.

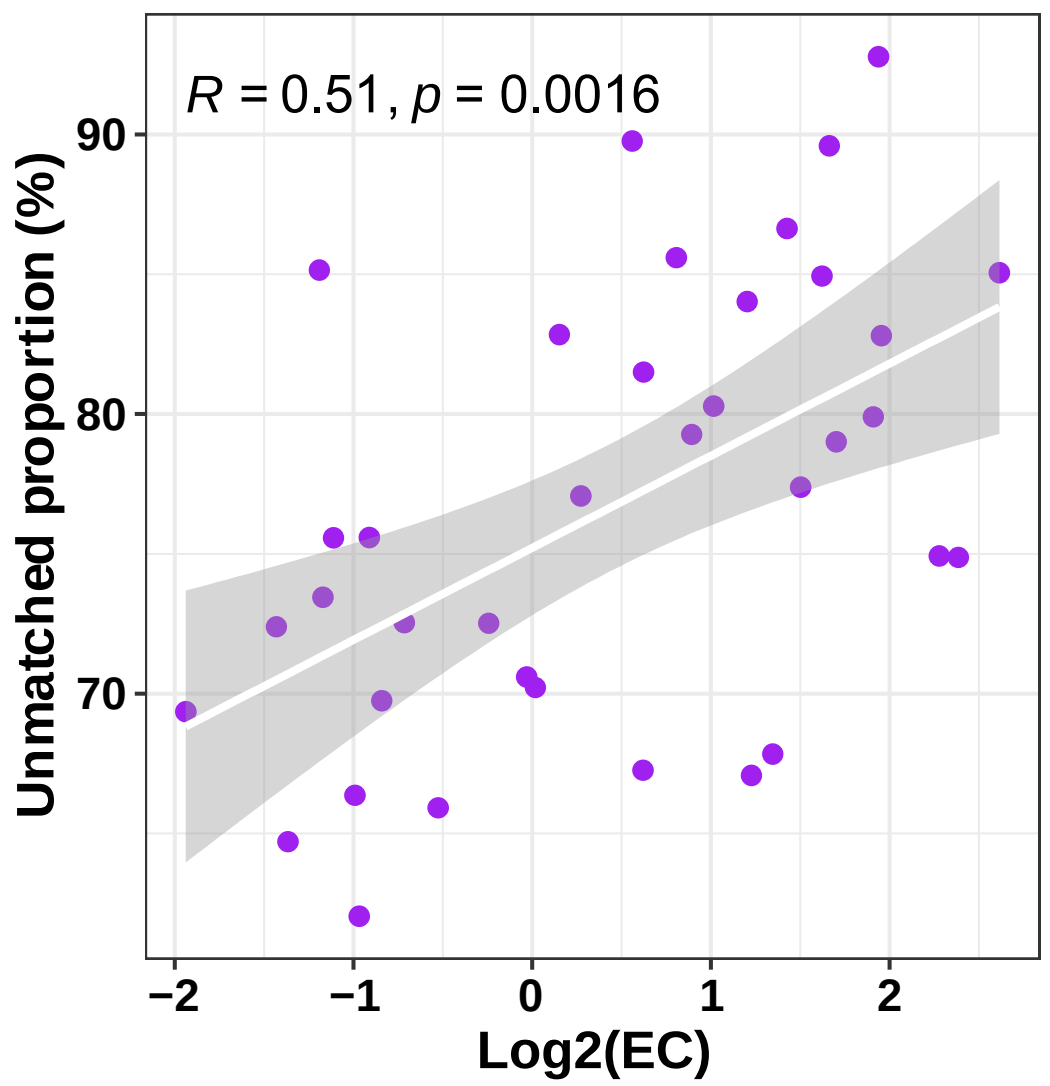

Figure S4 | Potential sources of unreliability in 16S-based average genome size (AGS) estimates in saline dataset. With mapping thresholds of > 99.5% identity and 100 % coverage, the proportion of GTDB-unclassified bacterial taxa increases with soil salinity, reaching > 90% in hypersaline samples, where AGS estimates from 16S rRNA metabarcoding appear less reliable.

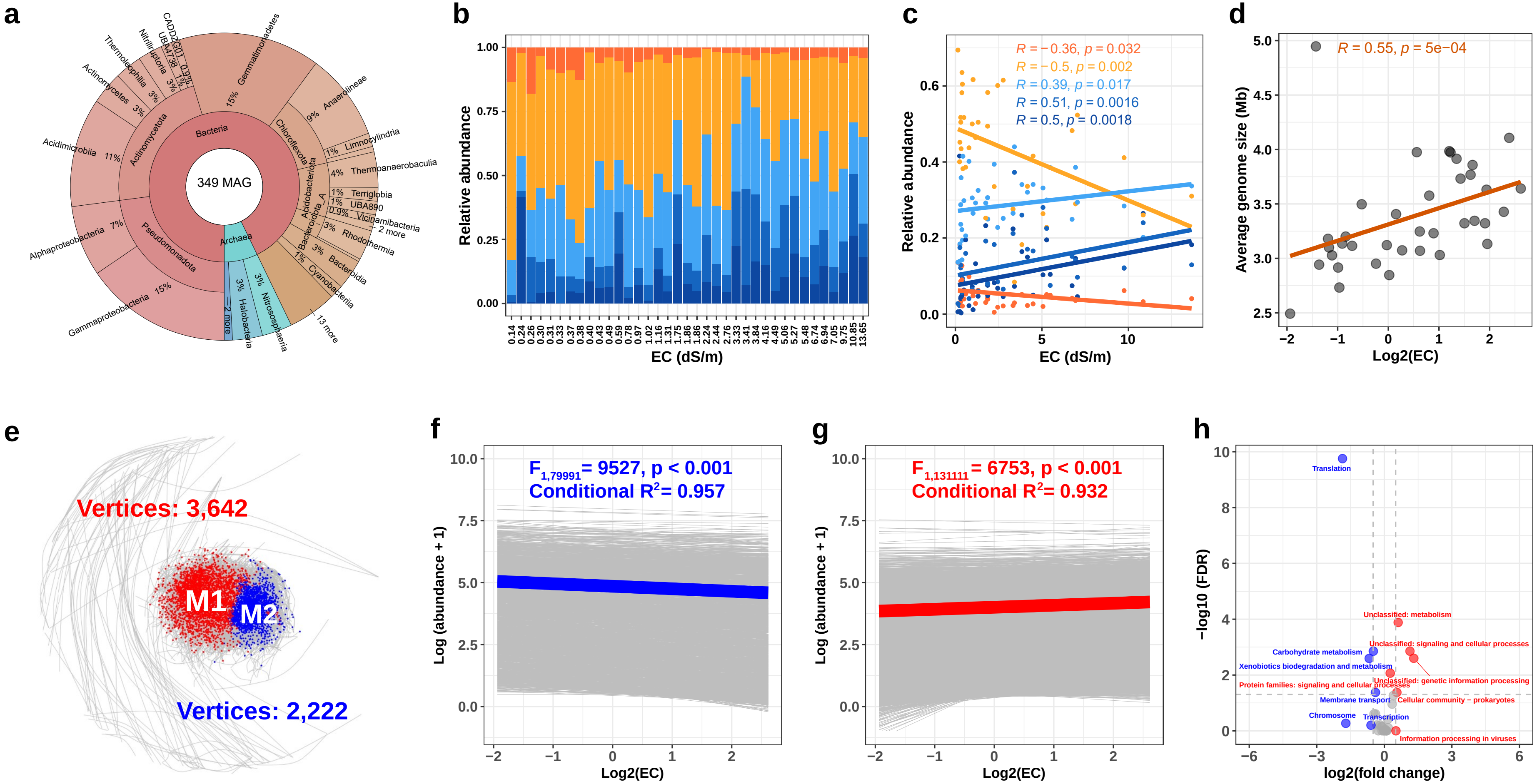

Figure S5 | Metagenome-assembled genomes (MAGs) and gene co-occurrence network modules cross-validate adjudication of conflicting average genome size (AGS) estimates in saline dataset. **a**, Overview of recovered MAGs. **b**, Relative abundance of MAGs grouped

by genome-size class. **c**, Smaller-genome MAGs decrease and larger-genome MAGs increase in relative abundance with higher salinity. **d**, MAG-based AGS increases with salinity. **e**, Gene (KO) co-occurrence network with two dominant modules: a larger saline module (red; 3,642 vertices) and a smaller non-saline module (blue; 2,222 vertices). **f–g,** Module–salinity associations (linear mixed-effects models, two-sided tests). The non-saline module (blue, **f**) decreases with soil salinity, whereas the saline module (**g**, red) increases with soil salinity. **h**, KEGG pathway enrichment (level 2) based on differential representation (two-sided tests). The saline module is enriched for signal and cellular processes, whereas the non-saline module is enriched for translation, xenobiotics biodegradation, and carbohydrate biosynthesis

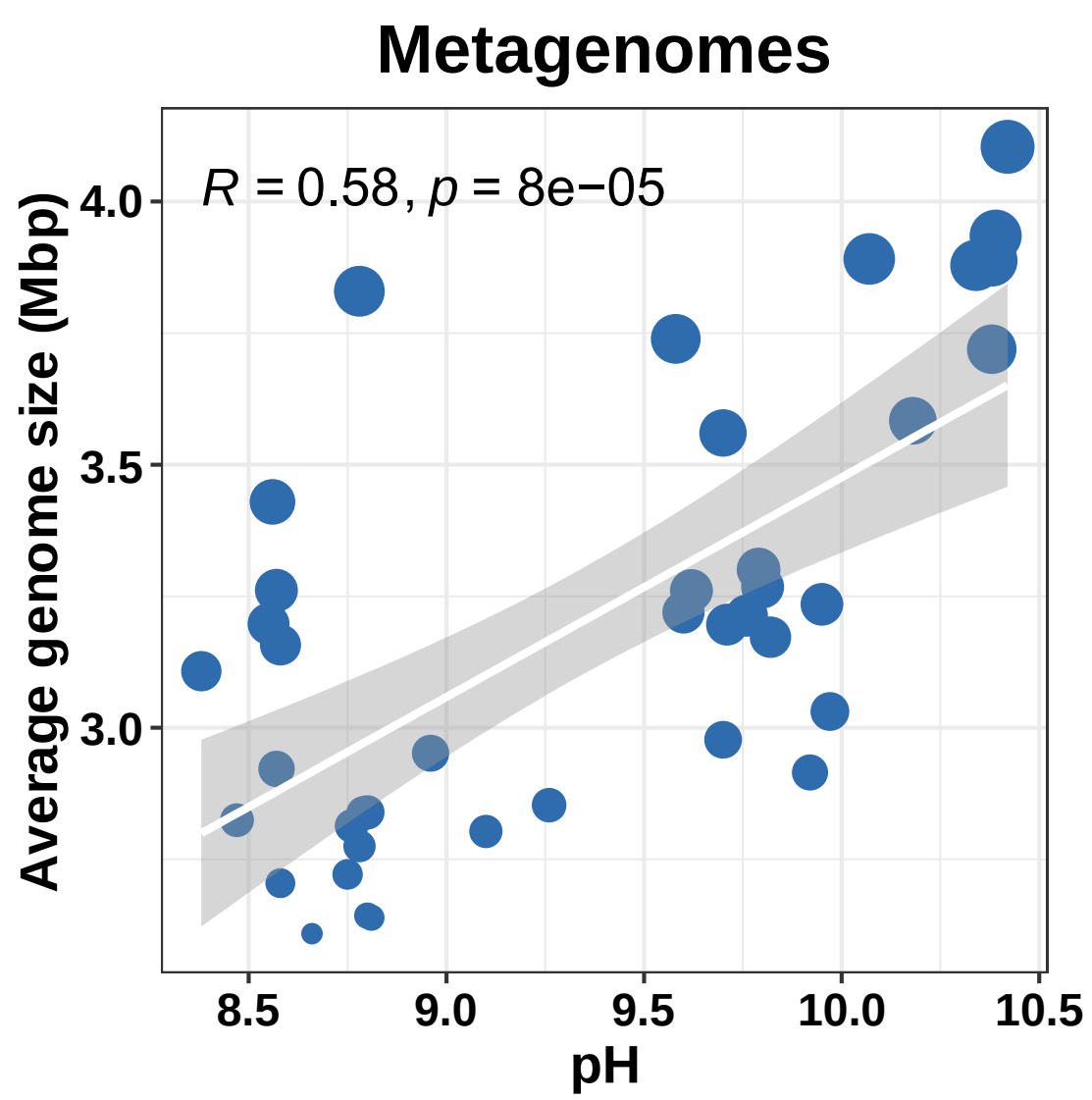

Figure S6 | Metagenomic AGS calculated based on reads remapped to prokaryotic contigs that identified by Kraken 2 in the alkaline dataset.

References

1 Ma, B., Lu, C., Wang, Y. *et al.* A genomic catalogue of soil microbiomes boosts mining of biodiversity and genetic resources. *Nature Communications* **14**, 7318 (2023), https://doi.org/10.1038/s41467-023-43000-z
